# Influence of Surface Chemistry of Fiber and Lignocellulosic Materials on Adhesion Properties with Polybutylene Succinate at Nanoscale

**DOI:** 10.3390/ma16062440

**Published:** 2023-03-18

**Authors:** Carlos Marcuello, Brigitte Chabbert, Françoise Berzin, Nicolas B. Bercu, Michael Molinari, Véronique Aguié-Béghin

**Affiliations:** 1 Université de Reims Champagne Ardenne, INRAE, FARE, 51097 Reims, France; 2 Université de Reims Champagne Ardenne, LRN, 51100 Reims, France; 3 Université de Bordeaux, CNRS, INP, CBMN, 33600 Pessac, France

**Keywords:** intermolecular interactions, plant fibers, lignocellulosic films, polybutylene succinate (PBS), atomic force microscopy, force spectroscopy, functionalization, infrared spectroscopy

## Abstract

The production of bio-based composites with enhanced characteristics constitutes a strategic action to minimize the use of fossil fuel resources. The mechanical performances of these materials are related to the specific properties of their components, as well as to the quality of the interface between the matrix and the fibers. In a previous research study, it was shown that the polarity of the matrix played a key role in the mechanisms of fiber breakage during processing, as well as on the final properties of the composite. However, some key questions remained unanswered, and new investigations were necessary to improve the knowledge of the interactions between a lignocellulosic material and a polar matrix. In this work, for the first time, atomic force microscopy based on force spectroscopy measurements was carried out using functionalized tips to characterize the intermolecular interactions at the single molecule level, taking place between poly(butylene succinate) and four different plant fibers. The efficiency of the tip functionalization was checked out by scanning electron microscopy and energy-dispersive X-ray spectroscopy, whereas the fibers chemistry was characterized by Fourier-transform infrared spectroscopy. Larger interactions at the nanoscale level were found between the matrix and hypolignified fibers compared to lignified ones, as in control experiments on single lignocellulosic polymer films. These results could significantly aid in the design of the most appropriate composite composition depending on its final use.

## 1. Introduction

The production of bio-based composites with enhanced characteristics has gained attention since governments make strong efforts to minimize the scarce fossil fuels being consumed [1]. In this context, the use of plant bast fibers (flax, hemp, jute, etc.) to replace glass or carbon fibers has significantly increased over the last two decades, due to their physicochemical properties, notably their low density and mechanical performance [2]. These fibers can be mixed with bio-sourced thermoplastic matrices such as polylactide (PLA), poly(hydroxyl alcanoate) (PHA), poly(butylene succinate) (PBS), or poly(butyrate adipate terephthalate) (PBAT) to obtain bio-based composites. The mechanical performances are related to the properties of the fibers and that of the matrix (Young’s modulus, aspect ratio, fiber composition, etc.), as well as to the quality of the interface between the matrix and the embedded material. A previous research study [3] showed that the polarity of the matrix played a key role in the mechanisms of fiber breakage during processing, as well as on the final properties of the composite. Moreover, these properties were different for a polar PBS matrix with hemp and flax fibers of similar dimensions, indicating different interactions between the fibers and the matrix. A recent study explored the correlations between the interfacial properties measured by micro-droplet tests and the composite tensile properties measured at the macroscale of flax-fiber-reinforced biodegradable thermoplastics [4]. Both mechanical and interfacial tests revealed that the adhesion of flax fibers is better with a biodegradable polymer (PBS, PHA, and PLA) than with a polypropylene (PP) matrix These adhesion properties could be evidenced at the nanometric scale by using atomic force microscopy (AFM). AFM consists of a flexible lever ended by a sharp tip which taps the external sample surface [5]. AFM is capable of acquiring the morphology of the scanned features with extremely high accuracy [6]. Then atomic force microscopy based on force spectroscopy (AFM-FS) detects the flexion of the lever, which follows the Hooke’s law with picoNewton sensitivity once the AFM probe is properly calibrated [7]. Specific intermolecular interactions can be achieved if the AFM tip and the sample surface are then tethered with the biomolecules or polymers of interest [8,9]. Thus, AFM is considered to be a multiparametric technique to assess multiple physicochemical properties at the nanoscale level [10]. The PBS matrix [11,12], lignocellulosic films [13], and composites reinforced by plant fibers [14,15] have already been studied by AFM imaging, but little work has been carried out in the field of AFM-FS. For this later method, the functionalization of AFM tips via a reproducible way is challenging and requests strong chemical knowledge of all steps involved in this process [16]. Notably, the results of intermolecular adhesion forces measured by AFM-FS between AFM levers functionalized with cellulose nanocrystals and the surface of composites have shown that cellulose nanocrystals display a stronger interaction with PBS than with the PP matrix [3].

Among plant fibers, bast fibers are thick-wall sclerenchyma cells located in the bark tissue of plants such as hemp, flax, jute, or kenaf. Despite some common morphological traits, chemical composition shows large variations depending on the developmental stage and botanical origin [17,18,19]. Flax and hemp fibers are hypolignified (less than 5% cell wall) in contrast to jute and kenaf fibers that contain 11–16% lignin [20,21,22,23]. Moreover, hemp and flax fiber are cellulose rich, ending in a highly crystalline structure [24,25].

In the present study, we hypothesized that variations in the surface chemistry of the fibers impact on their adhesion properties with composite matrix. AFM tips were functionalized with the PBS matrix by chemical coupling prior to the quantitative characterization of intermolecular adhesion forces with lignocellulosic polymer films and plant fibers longitudinal surfaces at the nanoscale. This comparison performed both on single lignocellulosic polymer films and on fibers with various lignin contents confirms that PBS exhibits the best adhesion on hypolignified materials.

## 2. Materials and Methods

### 2.1. Single Lignocellulosic Polymer Films and Bast Fibers

Lignocellulosic films were obtained as a single-polymer system, using glucomannan (GMK, low viscosity, Megazyme, Wicklow, Ireland), xylan (XYL) extracted from oat spelt [26], cellulose nanocrystals (CNC), or lignin. CNCs were isolated and purified from bleached ramie fibers (Stucken Melchers, Bremen, Germany) [27]. Stock solutions of GMK, XYL (10 g L^−1^), or CNCs (18 g L^−1^) were cast on Petri dish (43 cm^2^) and dried at ambient air for 24 h. Films thickness was measured using a dial thickness gauge (Käfer GmbH, Villingen, Germany), and the mean value was approximately 30 µm ± 2 µm for each film. Lignin-coated films were prepared from organosolv lignin solution (extracted from maize stalks (*Zea mays* L.) at 5 g L^−1^ in aqueous dioxane (9/1, *v*/*v*), and 30 μL of this solution was spin-coated onto silicon wafers (area of 1 cm^2^), using a commercial spin-coater (Specialty Coating Systems Inc., SCS P-6708/6712, Indianapolis, IN, USA) at 4000 rpm, as detailed in [28]. Prior to the coating, the silicon wafer was cleaned in freshly prepared Piranha solution for 30 min, exhaustively rinsed with ultrapure water, and dried before APTES (3-aminopropyltriethoxysilane, Sigma-Aldrich-Merck, Saint-Quentin-Fallavier, France) deposition in stream nitrogen gas to promote the adhesion of the lignin layer. The lignin-coated wafers were then heated in vacuum at 70 °C for 1 h to remove the last solvent molecules from the coated film.

Plant fibers (jute, kenaf, flax, and hemp fibers) were provided by Fibres Recherche Développement (FRD, Troyes, France). These fibers were stored in room conditions prior the AFM-FS measurements.

### 2.2. Infrared Spectroscopy

Plant fibers and lignocellulosic polymer films were analyzed by mid-infrared spectroscopy, using a Nicolet 6700 Thermo Electron FTIR spectrometer in attenuated total reflection (ATR-IR) mode equipped with diamond crystal that allows for the surface analysis of sample through a 2 µm effective depth. For each type of sample, 4 records were performed in the range of 1800–780 cm^−1^. Each record corresponds to an average of 32 scans with a spectral resolution of 4 cm^−1^. The baseline of the spectra was corrected by using OMNIC software and normalized by applying a correction factor 1000/(A1800–780 cm^−1^), where A1800–780 cm^−1^ is the area of the spectra between 1800 and 780 cm^−1^.

### 2.3. AFM Tip Functionalization with Poly(butylene succinate) (PBS)

The AFM tips (DNP, Bruker Probes, Camarillo, CA, USA, with nominal spring constant of 0.35 N m^−1^ and nominal frequency of 65 kHz) were initially washed in chloroform and dried with a nitrogen flux. Then 3-aminopropyltriethoxysilane (APTES) and Hunnig’s base (N-diisopropylamine) (Sigma-Aldrich-Merck, Saint-Quentin-Fallavier, France) were added in a ratio of 3:1 in volume for the amino functionalization of the AFM tip (Supplementary Figure S1a), as indicated elsewhere [29]. The reaction took place for 2 h under a nitrogen atmosphere. The AFM tip remained under the same conditions for 24 h before following the next chemical steps. Prior to PBS linkage, aminofunctionalized tips were chemically modified by adding a heterobifunctional linker molecule (3-maleimidopropionic acid N-hydroxysuccinimide ester, Sigma-Aldrich-Merck, France). Then 1 mg of this linker was dissolved in 470 µL of chloroform (Sigma-Aldrich-Merck, France) and 30 µL of triethylamine (Sigma-Aldrich-Merck, France). Aminated AFM tips were reacted with the linker reagent (2 mg mL^−1^) for 2 h at room temperature inside a polytetrafluoroethylene (PTFE) beaker (Fisher Scientific, Illkirch-Graffenstaden, France). The amine groups from the AFM tip reacted to the maleimide end from the linker molecule, forming stable amide bonds (Supplementary Figure S1b). Then the AFM tips were washed in chloroform three times for 10 min in order to release the excess of non-reacted linker molecules. In parallel, 1 mg of 3-(2-pyridyldithio)propionyl hydrazide (PDPH, Sigma-Aldrich-Merck, France) tether molecule was incubated with 1 mL of 4.4 mM tris-2-carboxyethyl)phosphine (TCEP, Sigma-Aldrich-Merck, France) dissolved in ethylenediaminetetraacetic acid (EDTA, Sigma-Aldrich-Merck, France) at a pH of 7.4 and ambient conditions. The maleimide hydrolysis and then the reaction stereospecificity were prevented by using a pH value under 8.0. The reductive TCEP agent converted the protective pyridyldithio groups from PDPH to active sulfhydryl moieties, thus rendering propanethiol hydrazide (Supplementary Figure S1c). Prior to the final functionalization step, the AFM tips were washed three times for 5 min with 100 mM TCEP solution to equilibrate the working pH and incubated with the previously propanethiol hydrazide solution for 2 h at room temperature inside a PTFE beaker. Thus, reactive hydrazone moieties were exposed at the external side of the AFM tip (Supplementary Figure S1d). Then PBS (NaturePlast, Ifs, France) stock solution was prepared by dissolving 1 mg PBS in 1 mL of dichloromethane at 50 °C for 2 h. Finally, the AFM tips were washed three times again with 100 mM TCEP solution and poured into a previously prepared PBS stock solution diluted with ultrapure toluene (Sigma-Aldrich-Merck, France) and left overnight at room temperature, in darkness conditions (Supplementary Figure S1e). The AFM tips functionalized with PBS were gently washed with toluene three times before their thorough preservation in clean AFM boxes before carrying out force spectroscopy measurements.

### 2.4. SEM Observation and EDXS Measurement of PBS Functionalized Tip

The efficiency of the AFM tip functionalization with PBS was checked by scanning electron microscopy (SEM), using a JEOL (Tokyo, Japan) JSM-7900F setup. SEM images were recorded from the secondary electrons at primary beam energies of 1.5 keV. SEM setup was coupled with UltiMax 170 mm^2^ Energy dispersive X-Ray spectroscopy (EDXS) setup. EDXS was used to identify the elemental composition of the AFM tip apex after the functionalization procedure with PBS.

### 2.5. Atomic Force Microscopy Imaging

The topography of lignocellulosic films (CNCs, GMK, XYL, and LIG) and plant fibers was acquired by PeakForce tapping mode in air to check the integrity and the homogeneity before spectroscopy force experiments. AFM imaging was carried out at 25 °C and 45 ± 0.3% relative humidity (RH), using a Multimode-8 AFM setup (Bruker, Santa Barbara, CA, USA), which was coupled with a Wetsys system RH generator (Setaram Instrumentation, Caluire-et-Cuire, France). The value of the relative humidity was monitored during image acquisition by a Tinytag device (Gemini Data Loggers, Chichester, UK). ScanAsyst Air probes (Bruker Probes, USA) with a nominal spring constant of 0.4 N m^−1^, a nominal frequency of 70 kHz, and a tip radius of 2 nm were used to acquire topography information necessary to calculate the root mean square average (Rq) roughness parameter of the lignocellulosic films and the plant fibers, as indicated elsewhere [28]. At least three representative AFM images from different locations were analyzed. All AFM images were analyzed while following the same procedure and settings in order not to induce Rq deviations based on data handling.

### 2.6. Force Spectroscopy Measurements

The intermolecular forces between the functionalized tips with PBS and lignocellulosic materials (films and plant fibers) were investigated using the AFM equipment, as described in the previous section, but operating with force−volume (F−V) mode in air. This mode measures the real-time mapping of local areas, which allows for the quantification of adhesion forces extracted from force–distance curves, which were analyzed as indicated elsewhere [28]. Longitudinal portions of the plant fibers (2 cm length) were cut with a scalpel, and their extremities were fixed on an iron disk surface, using a 1–2 µL drop of a fast UV-light curing adhesive (DYMAX, Torrington, USA). All experiments were carried out under a controlled relative humidity at 45 ± 0.3% at 25° C. Topography and adhesion force maps were simultaneously acquired using similar operational settings: 200 nN s^−1^ as the load rate, 500 nm as ramp size, 0 s as the retract delay, and 5 μm × 5 μm as the scan size for all samples, with the exception of CNCs film, where the scan size was defined as 1 μm × 1 μm. Then 48 ramps per line (2304 force curves) and 60 ramps per line (3600 force curves) were acquired for the studied single lignocellulosic polymer films before applying this methodology on the longitudinal surfaces of plant fibers. The topography of the functionalized AFM tips with PBS was recorded before and after the F−V acquisition to ensure proper PBS attachment. Histograms from the adhesion data were fitted by a Gaussian distribution. Histogram bin size was defined at 0.5 nN for all force distributions.

### 2.7. Statistical Analysis

One-way analysis of variance (ANOVA) calculations were used for the adhesion forces recorded between PBS functionalized tips with respect to the studied lignocellulosic films and the plant fibers. Additionally, to ascertain the significantly differences of the groups, a Turkey–Kramer post hoc test was carried out in combination with the r-Pearson test. Significant differences were assumed with a significance level greater than 95% (*p* < 0.05).

## 3. Results and Discussion

### 3.1. Infrared Characterization of Lignocellulosic Materials

**Figure 1:**
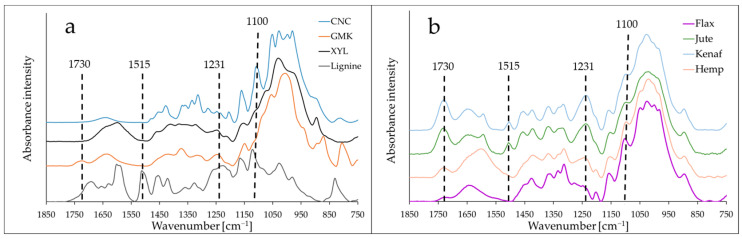
ATR infrared spectra of lignocellulosic polymer films (**a**) and plant fibers (**b**). The vertical lines indicate four main band variations observed that are specific to the composition of films and fibers in polymers (cellulose, hemicelluloses, and lignin).

The ATR-IR spectra of the different polymer films and the plant fibers (kenaf, jute, flax, and hemp) show large variations in intensity at specific bands according to their surface composition (Figure 1). Between cellulose (CNC) and hemicellulose (GMK and XYL) films, the main variations were observed at 1730 cm^−1^ (corresponding to acetyl group (C=O) for hemicellulose) and at 1160 and 1100 cm^−1^ corresponding to the glycosidic bonds (C-O-C) and secondary alcoholgroups (C-O stretching), respectively, for cellulose. The main differences between lignin and polysaccharidic films were observed at 1515 cm^−1^ (C=C aromatic skeletal) and at 1231 cm^−1^, corresponding to the C-O stretching in aromatic rings and in xylan [30]. For the ATR spectra of bast fibers, three main variations were observed at 1730 cm^−1^, 1506 cm^−1^, and 1231 cm^−1^ between (flax and hemp) and (kenaf and jute) fibers. No significant band characteristic of lignin (1506 cm^−1^) was found for flax and hemp (hypolignified) fibers in contrast to jute and kenaf. Likewise, the band intensity at 1231 cm^−1^ for jute and kenaf was approximately twice that of flax and hemp. Regarding the low intensity of polysaccharide regions, albeit the high intensity for cellulose band (1100 cm^−1^), other differences in intensity of bands were related to non-cellulosic polysaccharides (1730 cm^−1^), as jute and kenaf contain more hemicelluloses (as mainly xylan) than flax or hemp. In consequence, the calculated lignin/cellulose intensity ratio, 1506/1100, varied from 0.01 to 0.03 (flax and hemp) and from 0.14 to 0.22 (jute and kenaf), and the ratio 1231/1100 corresponding to aromatic and hemicelluloses bands increased in the following order: 0.18 (flax), 0.31 (hemp), 0.56 (jute), and 0.60 (kenaf) (Table 1). These results are in agreement with the published data indicating that jute and kenaf contain more lignin than flax and hemp [19]. In addition, the content and type of hemicelluloses varies according to the plant fibers [31]. The lignified cell walls in wood and some bast fibers (kenaf, jute) contain xylan as the main hemicellulose, whereas cellulose rich fibers as flax contain more pectin, galactans, and hemp contain more glucomannan [20].

### 3.2. Tip Functionalization by PBS

**Figure 2:**
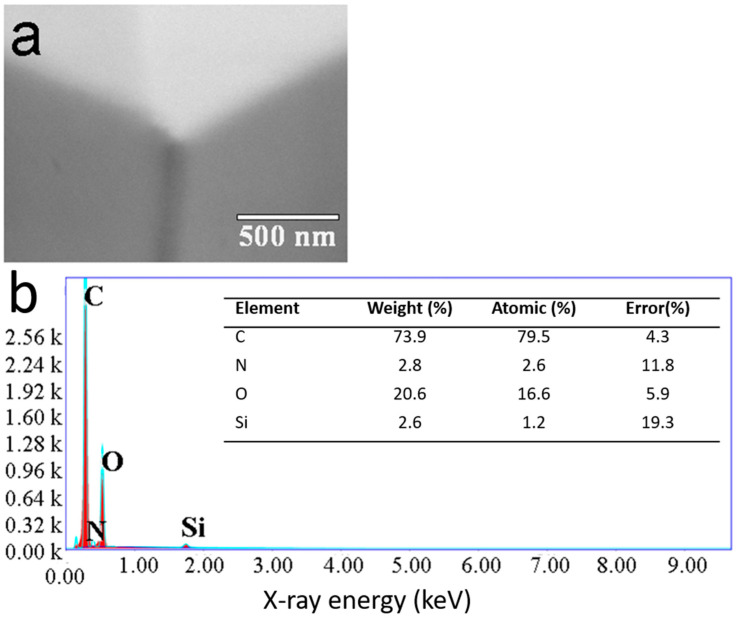
(**a**) SEM image of the AFM tip apex after the PBS functionalization procedure. (**b**) EDXS spectrum of the selected region and the subsequent elemental analysis.

The efficiency of AFM tip functionalization with PBS was checked by SEM and EDXS after the functionalization procedure. Looking at the SEM image, it seems that the procedure leads to the coating of the tips with a homogeneous layer (Figure 2a). To be sure that a PBS layer is effectively deposited on the silicon nitride tip, EDXS measurements (Figure 2b) were performed by focusing the beam on the tip apex. The EDXS spectrum demonstrated the existence of carbon (C), nitrogen (N), oxygen (O), and silicon (Si) atoms on the tip. The main element observed was C (79.5%), followed by O (16.6%), N (2.6%), and Si (1.2%), which is in good agreement with the chemical composition of PBS (10 C = 120 g moL^−1^, i.e., 71.4%; 3 O = 48 g moL^−1^, i.e., 28.6%) [32] and confirmed the deposition of PBS on the tip. The low percentages of silicon and nitrogen also evidenced (<3%) are coming from the Si_3_N_4_ bare tips. As it was shown that the beam penetration depth on gold surfaces ranged from 20 nm at 2.0 keV to 3 nm at 0.5 keV, respectively [33], we could assume that, with an EDXS voltage of 1.5 keV and the weakness of the signal coming from the bare tip, the thickness of PBS is in the range from 10 to 20 nm.

### 3.3. Adhesion Properties between the PBS Functionalized AFM Tips and Lignocellulosic Films

**Figure 3:**
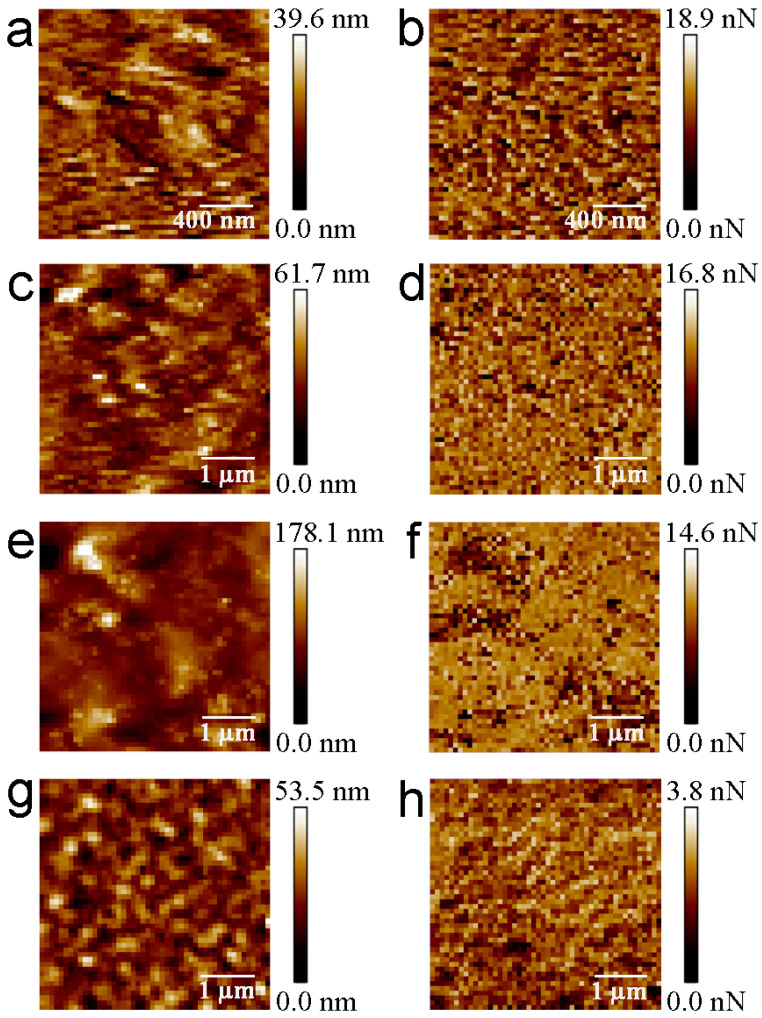
Force–volume experiments between PBS functionalized tips and CNCs (**a**,**b**), GMK (**c**,**d**), XYL (**e**,**f**), and LIG (**g**,**h**). Topography and force maps are represented on the left and right side, respectively.

Different single-polymer films (CNC, GMK, XYL, and LIG) were characterized. The AFM images were acquired in several areas of each film to ensure the homogeneity of the exposed external surface. The mean values of the height and roughness were analogous to those of previous works, 6.2 ± 0.2 nm, 9.1 ± 0.7 nm, 23.6 ± 1.8 nm, and 10.0 ± 0.5 nm, respectively [28]. Then force spectroscopy measurements were performed between the PBS functionalized tip and the aforementioned lignocellulosic polymers in order to accurately determine the intermolecular interactions displayed between PBS and the main components of the plant cell walls. The F−V mode allows for the simultaneous acquisition of both topography and adhesion force maps (Figure 3).

Table 2 summarizes the adhesion force values exerted between PBS and the four tested lignocellulosic polymer films. It is remarkable that the established interaction between the PBS tip and lignin films is notably lower (3.4 ± 0.1 nN) than the adhesion force observed with cellulose and hemicellulose films (10.5 ± 1.4 nN, 11.7 ± 0.7 nN, and 9.0 ± 0.6 nN for PBS:CNCs, PBS:GMK, and PBS:XYL, respectively).

**Figure 4:**
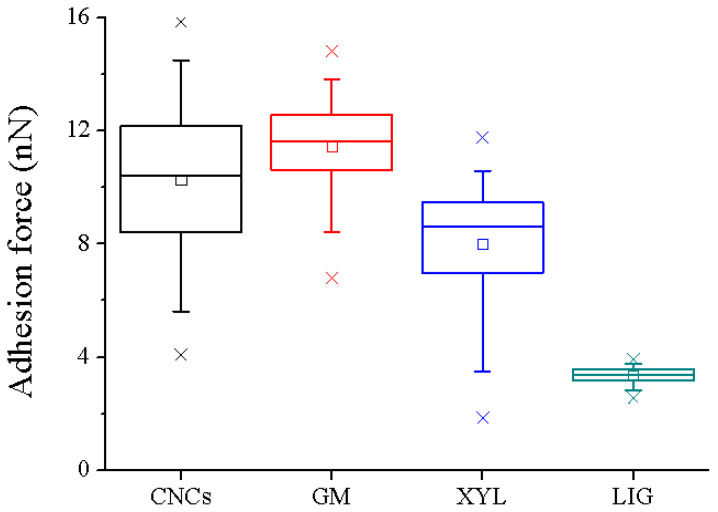
Box plot of the statistical analysis of the force distributions obtained between PBS functionalized tips and the different films.

Thus, the adhesion forces are nearly three times lower when the PBS interacts with lignified surfaces. This finding is not unexpected since lignin and PBS molecules present a large amount of hydrophobicity due to their phenolic groups [34] and the presence of butenoic acid units, which may induce hydrophobicity in the copolymers [35], respectively. The statistically significant differences observed for the adhesion forces between PBS with lignin and the rest of the analyzed films are shown in Figure 4. Moreover, the adhesion force between PBS tip and CNCs film is about 10–11 nN, which is considerably lower than the force values measured between AFM levers functionalized with CNCs and PBS matrix surfaces, being nearby 16 nN [8]. This contrast can be due to the different contact surface areas between two solid surfaces for the previous methodology [8] and between a solid surface and a sharp hemisphere (functionalized AFM tip) in this study.

The aim of this set of experiments is to gather further insights into the extension of the forces involved in the binding of PBS functionalized tip with single lignocellulosic polymer films. This information will significantly aid in our understanding of the interactions taking place between the same PBS tip and the longitudinal surfaces of native blast fibers. The upcoming subsection concerns F–V experiments with PBS tips on the four tested bast fibers.

### 3.4. Adhesion Properties between the PBS Functionalized AFM Tip and the Surface of Bast Fibers

**Figure 5:**
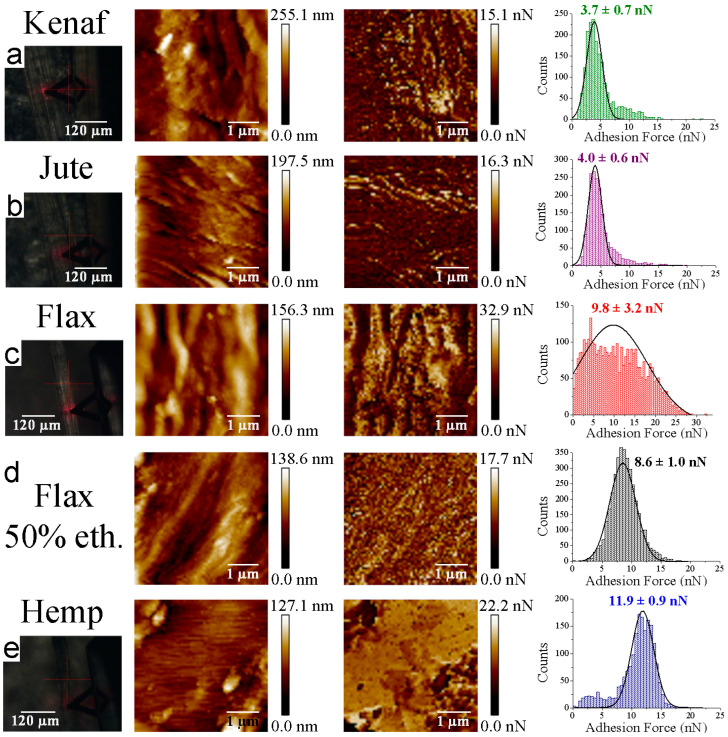
Force–volume experiments conducted between PBS functionalized tip and (**a**) kenaf, (**b**) jute, (**c**) flax, (**d**) flax washed with 50% ethanol, and (**e**) hemp fibers. From left to right side: zenithal optical microscope view, F–V topography map, F–V force adhesion map, and the histogram force distribution for each assessed plant fibers.

The interaction forces of four different bast fibers (kenaf, jute, flax, and hemp) were measured by AFM-FS, using PBS functionalized tip (Figure 5). The zenithal view of the optical microscope coupled to the AFM setup allowed us to determine the accurate position of the PBS tip on the top of the plant fiber external surface. The roughness values of kenaf, jute, flax, and hemp fibers were equal to 39.7 ± 2.7 nm, 35.1 ± 13.7 nm, 76.2 ± 24.5 nm and 61.5 ± 16.2 nm, respectively (Supplementary Figure S2). The force spectroscopy experiments were successfully conducted for all tested samples. For the different samples, similar Gaussian distributions of the adhesion forces were obtained, except for the flax fibers, which exhibit a large distribution (Figure 5c). Thus, to improve the measurements, flax fibers were washed with 50% ethanol (two incubations for 2 min each, followed by drying under nitrogen flux) to remove dust prior to measurement of adhesion forces with PBS (Figure 5d). A narrower Gaussian distribution was then obtained following this cleaning step. This quick washing step was applied only on flax fibers, as broad force distributions were observed only in this experimental condition. The other plant fibers did not require us to proceed with this cleaning procedure based on their initial narrow force distributions. If some weak changes cannot be excluded after the quick washing step (the objective of which was to remove some remnants of microorganisms and/or outer tissues of the flax stem (epidermis), as flax is mechanically extracted from the stem after a first-step retting on the field), the average values of flax and ethanol-washed flax are 9.8 ± 3.2 nN and 8.6 ± 1.0 nN, respectively, which are so much higher than the values close to 4.0 nN obtained for jute and kenaf.

**Figure 6:**
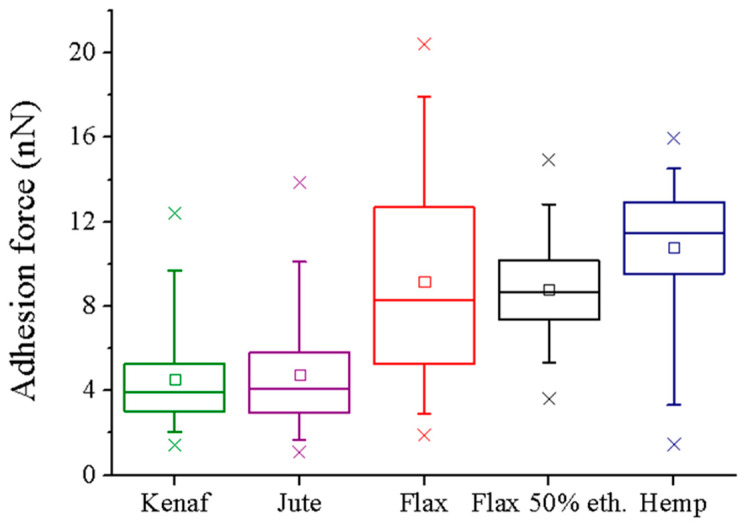
Statistical analysis of the rupture forces obtained between PBS functionalized tip and the longitudinal surface of plant fibers.

Representative force curves are shown in Supplementary Figure S2. Since the rupture bonds which are forming a particular complex are stochastic events [36], a considerably huge volume of force curves is required to accurately calculate the most probable unbinding force through Gaussian distributions, as indicated in Figure 5 (right side). The results demonstrated that lignified bast fibers (kenaf and jute) exhibit lower interaction forces with PBS functionalized tip (3.7 ± 0.7 nN and 4.0 ± 0.6 nN for kenaf and jute fibers, respectively) than hypolignified fibers (8.6 ± 1.0 nN and 11.9 ± 0.9 nN for flax and hemp fibers, respectively). Furthermore, flax fibers washed with 50% ethanol (8.6 ± 1.0 nN) did not present strong differences regarding native flax samples. Even if the ultra-sharp AFM tips radius (with nominal radius of 2 nm) used can be increased after functionalization (no more than 20–30 nm, as evidenced by SEM images), the mean contact between the tip and a rough/flat surface is similar. The only difference of the data recorded in the case of sharp tips with rough/flat surfaces is the scattering of the gathered force results [37]. Moreover, the differences observed between the adhesion forces between PBS and lignified/hypolignified fibers are statistically significant, as depicted in Figure 6. It could be noted that, for the hemp sample, the number of measurements was smaller because of the difficulty in acquiring reliable data on different regions. This fact could be linked to larger unspecific interactions between the PBS tip and the external hemp fiber surface which led to instabilities during force data acquisition.

For the different fibers, the data are in good agreement with the results obtained with single lignocellulosic polymer films. Such variation between lignified and hypolignified fibers can be explained by the polarity of PBS. The nature of this material is partially hydrophilic after its polymerization synthesis process. Indeed, PBS polymer chain extension is formed by the polycondensation reactions of the direct esterification of a dicarboxylic acid and a diol, which are very polar components. For this reason, a polar material such as PBS can establish strong interactions with other high-polar surfaces, such as hypolignified fibers (such as flax and hemp), which mostly contain cellulose and non-cellulosic polysaccharides (pectins and hemicelluloses), as shown by ATR-IR (Table 1).

These results make a significant contribution to improving our understanding of how the PBS matrix interacts with different plant fillers. Playing with the plant fiber sources and their lignin content, it may be possible to design composites with their mechanical performance tuned to the intended use. Some previous efforts have been made in this field, such as the continuous attempt of improving the interfacial adhesion properties between PBS and lignin [38], which may be of further interest for many industrial applications, such as packaging [39], tissue engineering [40], or the implementation of more effective antibacterial treatments [41,42], among others.

## 4. Conclusions and Perspectives

The adhesion properties between a polar matrix (PBS) and four relevant bast fibers (kenaf, jute, flax, and hemp) were addressed for the first time at the single-molecule level. For it, AFM-FS measurements were carried out with coated PBS tips where the apex contacts the longitudinal external portion of the fibers, thus recording the force interaction between these surfaces under controlled conditions. The obtained results showed that PBS has higher adhesion forces with hypolignified fibers (flax and hemp) than with lignified ones (jute and kenaf). These findings were corroborated by AFM-FS control experiments on single lignocellulosic polymer films (cellulose, glucomannan, xylan, and lignin) and ATR-IR measurements of the surface chemical composition. Whereas cellulose and hemicelluloses, which mainly consist of hydroxyl groups, can establish more affinity with polar materials such as PBS, lignin is more hydrophobic than polysaccharides, thus significantly limiting the extension of bond formation with the matrix of interest. This fact evidences the strong impact of surface chemistry on matrix–filler adhesion properties.

Thus, future research on the adhesive properties between polymer matrices such as PLA, PHA, or PBAT and natural plant fibers could be extended by using this methodology. This will provide a more comprehensive view of the interfacial forces exerted between the polymer matrix and green-sourced biobased materials.

## Figures and Tables

**Table 1:** Calculated specific IR bands ratio in blast fibers.

Fibers	Specific IR Bands Ratio		
	1730/1100	1506/1100	1231/1100
Flax	0.06	0.01	0.18
Hemp	0.18	0.03	0.31
Jute	0.49	0.22	0.56
Kenaf	0.54	0.14	0.60

1730 cm^−1^ (acetyl group (C=O) of hemicelluloses and pectins), 1506 cm^−1^ (C=C aromatic skeletal of lignin), 1231 cm^−1^ (C-O stretching in aromatic rings and in hemicellulose), and 1100 cm^−1^ (secondary alcohol groups, C-O stretching of cellulose).

**Table 2:** Adhesion forces between PBS functionalized tip and lignocellulosic polymer films (nN). The abbreviations CNCs, GMK, XYL, and LIG refer to cellulose nanocrystals, glucomannan, xylan, and lignin, respectively.

CNCs	GMK	XYL	LIG
10.5 ± 1.4	11.7 ± 0.7	9.0 ± 0.6	3.4 ± 0.1

## Data Availability

Additional data are available upon reasonable request.

## Supplementary Materials

The following supporting information can be downloaded at https://www.mdpi.com/article/10.3390/ma16062440/s1, Figure S1: Schematic representation of the functionalization strategy followed to covalently tether PBS on AFM tips; Figure S2: AFM topography images of (a) kenaf, (b) jute, (c) flax, and (d) hemp fibers; Figure S3: Representative force curves of the studied plant fibers. Inset: zoom of the force curves depicted by a black arrow; Table S1: Roughness (Rq) analysis of the plant fiber surfaces (nm).
