# Supplementary material for: Influence of Surface Chemistry of Fiber and Lignocellulosic Materials on Adhesion Properties with Polybutylene Succinate at Nanoscale
Source: Materials (Basel). 2023 Mar 18;16(6):2440. doi: 10.3390/ma16062440 (PMC10056059; doi:10.3390/ma16062440)
Supplement: Supplementary file 1 [file materials-16-02440-s001.zip › materials-2230098-supplementary.pdf]

Supplementary data

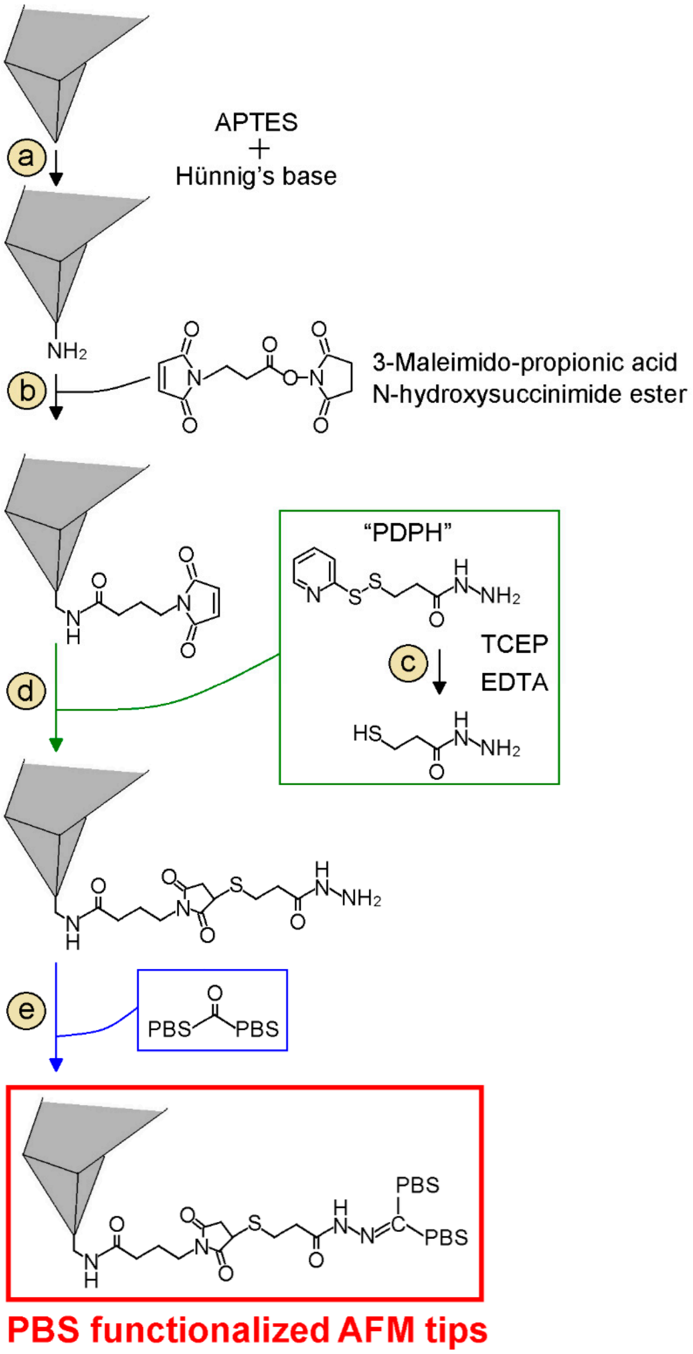

Figure S1. Schematic representation of the functionalization strategy followed to covalently tether PBS on AFM tips. (**a**) Aminofunctionalization procedure. (**b**) Reaction of aminated tips with the selected heterobifunctional linker to expose maleimide groups. (**c**) Activation of PDPH linker to exhibit reactive sulfhydryl groups. (**d**) These sulfhydryl groups react with the maleimide coming from the AFM tip to form thioether bonds. (**e**) Stable hydrazone bond (R_1-NH-N=C-R_2) formation to link PBS molecules into AFM tips through their ketone moieties. AFM-FS measurements can be devoted once the AFM tips are externally coated with PBS.

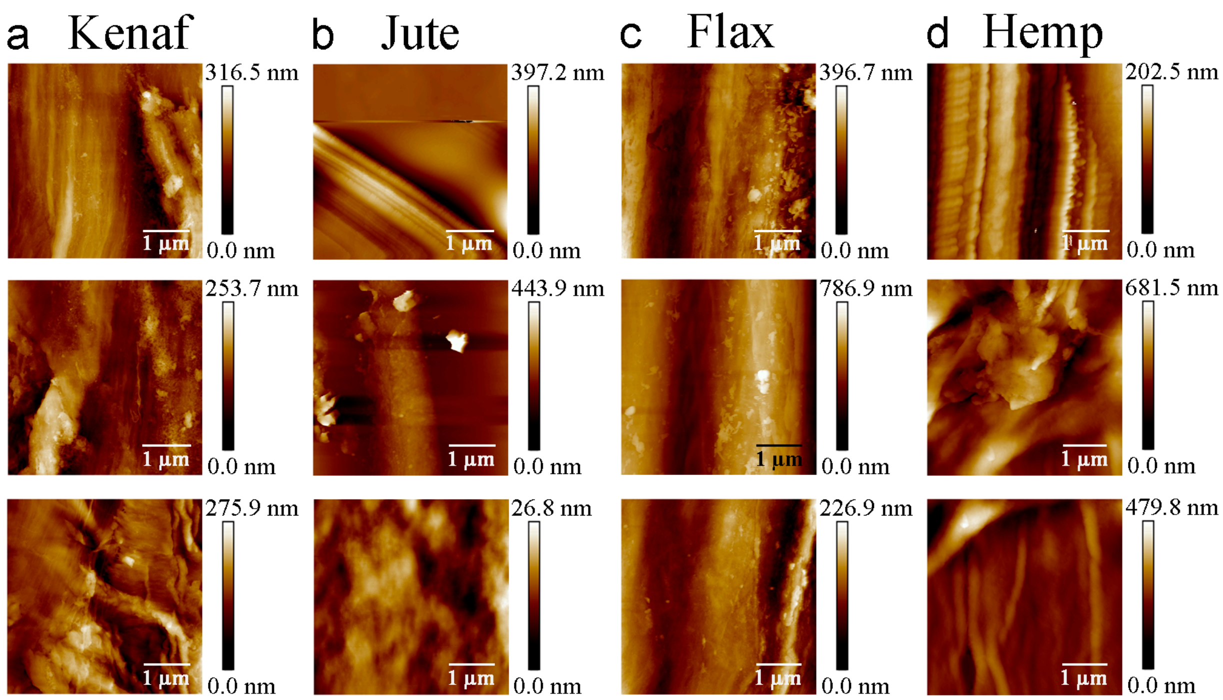

Figure S2. AFM topography images of (**a**) Kenaf, (**b**) Jute, (**c**) Flax and (**d**) Hemp fibers.

Table S1. Roughness (Rq) analysis of the plant fiber surfaces (nm).

	**Lignified fibers**		**Hypolignified fibers**	
	**Kenaf**	**Jute**	**Flax**	**Hemp**
**Area 1**	45.1	47.1	64.8	34.8
**Area 2**	34.3	54.4	130.0	97.7
**Area 3**	39.6	3.8	33.9	52.1
**Mean**	39.7 ± 2.7	35.1 ± 13.7	76.2 ± 24.5	61.5 ± 16.2

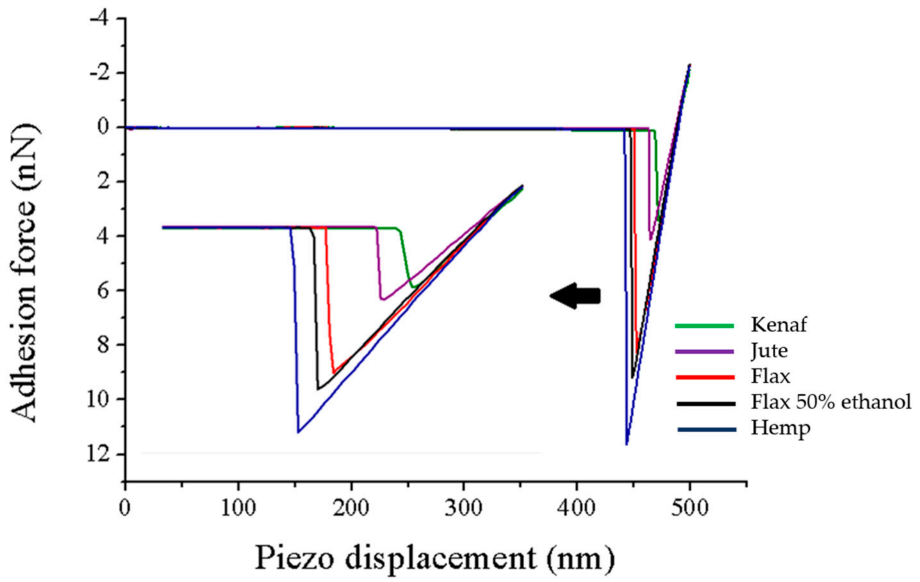

Figure S3. Representative force curves of the studied plant fibers. Insert: zoom of the force curves depicted by a black arrow.
